# Hypolipidemic Effects of Three Purgative Decoctions

**DOI:** 10.1093/ecam/nep178

**Published:** 2011-02-14

**Authors:** Sung-Hui Tseng, Ting-Yi Chien, Jiun-Rong Chen, I-Hsin Lin, Ching-Chiung Wang

**Affiliations:** ^1^School of Medicine, Taipei Medical University, 250 Wu- Xing Street, Taipei 110, Taiwan; ^2^School of Pharmacy, Taipei Medical University, 250 Wu-Xing Street Taipei 110, Taiwan; ^3^School of Nutrition and Health Sciences, Taipei Medical University, 250 Wu- Xing Street, Taipei 110, Taiwan; ^4^Committee on Chinese Medicine and Pharmacy Department of Health, Executive Yuan 6 Shuang-cheng Street, Taipei 104, Taiwan

## Abstract

In traditional Chinese medicine (TCM), purgation is indicated when a person suffers an illness due to the accumulation of evil internal heat. Obese individuals with a large belly, red face, thick and yellow tongue fur, constipation, and avoidance of heat are thought accumulates of evil internal heat, and they are also treated with purgatives such as *Ta-Cheng-Chi-Tang* (TCCT), *Xiao-Chen-Chi-Tang* (XCCT), and *Tiao-Wei-Chen-Chi-Tang* (TWCCT) by TCM doctors. In previous studies, our group found that TCCT has potent anti-inflammatory activity, and that XCCT is an effective antioxidant. Since rhubarb is the principle herb in these three prescriptions, we will first present a thorough review of the literature on the demonstrated effect (or lack of effect) of rhubarb and rhubarb-containing polyherbal preparations on lipid and weight control. We will then continue our research with an investigation of the anti-obesity and lipid-lowering effect of TCCT, XCCT, TWCCT, and rhubarb extracts using two animal models. TWCCT lowered the serum triglyceride concentration as much as fenofibrate in Triton WR-1339-treated mice. Daily supplementation with XCCT and TWCCT significantly attenuated the high-fat-diet-induced hypercholesterolemia in rats. In addition, TWCCT also significantly lowered the high-fat-diet-induced hypertriglycemia. Although feeding high-fat diet rats with these extracts did not cause loose stools or diarrhea or other deleterious effects on renal or hepatic function. None of these extracts lowered the body weight of rats fed on high-fat diet. In conclusion, the results suggest that XCCT and TWCCT might exert beneficial effects in the treatment of hyperlipidemia.

## 1. Introduction

In traditional Chinese medicine (TCM), obesity is classified into different types based on etiology; for example, stagnation of qi, blood, or phlegm; or a deficiency in the spleen or kidney energy. Obese individuals with a large belly, red face, thick and yellow tongue fur, constipation, and avoidance of heat are thought to have accumulated evil heat in the stomach. TCM doctors typically have used purgation to clear the evil heat in such individuals [1]. *Ta-Cheng-Chi-Tang* (TCCT), *Xiao-Chen-Chi-Tang* (XCCT), and *Tiao-Wei-Chen-Chi-Tang* (TWCCT) are the three famous purgative decoctions originally mentioned in *Shan han lun*, one of the most important Chinese medical books, written by Chang Chung-Ching (*∼*200 AD). These three purgatives are indicated when there is a need to purge internal evil heat that has raised symptoms like abdominal pain and distention, thirst, and fever [2]. Purgatives are also used to treat a type of obese person who has excessive evil internal heat. However, scientific research on the efficacies and mechanisms of these purgatives as anti-obesity agents is still very limited.

Rhubarb (Da Huang), the root of the *Rheum* plant species (Polygonaceae), is one of the most frequently employed herbs in treating obesity in TCM [3]. It is also the major constituent in TCCT, XCCT, and TWCCT. Rhubarb also appears in anti-obesity scientific research done by Western scientists. In fact, use of non-prescription laxatives is a commonly reported weight-loss behavior in Western society, especially among those who perceive themselves as being overweight [4, 5]. The prevalence of obesity is increasing, along with the demand for effective and safe anti-obesity agents, including herbal medicinal products [6]. Thus, the efficacy and safety of rhubarb and rhubarb-containing polyherbal preparations as anti-obesity and hypolipidemic agents should be substantiated. Therefore, we first conducted a review of literature by retrieving all the available scientific research from the following databases: Medline, Pubmed, Chinese Electronic Periodicals Service, and Wan Fang Data. Search terms included the following keywords in English and Chinese: Rhei, Rhei Rhizoma, rhubarb, *Rheum*, Da Huang, obesity, lipid, cholesterol, triglyceride, weight, high-fat diet, and Triton WR-1339. All literature pertaining to the efficacy of rhubarb and rhubarb polyherbal products in reducing body weight and lipids in human and animal studies was retrieved and appraised. We found that rhubarb and rhubarb-containing polyherbal preparations exhibited potential lipid- and body weight-lowering effects as reported in some animal studies, but the effects differed among studies as shown in Table 1 [7–14].

A summary of the human clinical trials examining the metabolic effect of these herbal preparations on body weight and lipid profiles is presented in Table 2 [15–22]. Overall, these studies reported the anti-obesity and hypolipidemic activities of three kinds of rhubarb extracts and seven rhubarb-containing polyherbal preparations. However, fewer than half of the collected studies revealed a significant efficacy in body-weight or lipid reduction. Demonstrated effects (or lack of effects) may depend on dosage and extract type used for a particular herb or herbs in poly-herbal preparations. The results suggest that the efficacy of rhubarb or rhubarb-containing polyherbal preparations on weight loss and lipid control warrants needs further investigation.

The development of many chronic diseases, such as coronary heart disease, diabetes mellitus, fatty liver disease, are closely related to obesity. Inflammation and excess oxidative stress contribute at least partially to the pathogenesis of these diseases [23–25]. Recently, we showed that TCCT, in addition to having potent purgative activity, also has a strong anti-inflammatory effect. We also showed that XCCT can protect the liver from oxidative stress [26, 27]. Hence, we postulated that these herbal purgative remedies may have potential value in the management of obesity and related conditions, such as hyperlipidemia. Therefore, we measured the anti-obesity and hypolipidemic activities of these three decoctions in rats fed with a high-fat diet and in Triton WR-1339-treated mice.

## 2. Methods

### 2.1. Preparation of Aqueous Extracts

Medicinal plants and materials used in the experiment included the root of *Rheum palmatum* L. (Polygonaceae), the bark of *Magnolia officinalis* Rehd. et Wils. (Magnoliaceae), the immature fruit of *Citrus aurantium* L. (Rutaceae), the root of *Glycyrrhiza uralensis* F. (Leguminosae), and *Mirabilitum* (mirabilite, crystals of sodium sulfate, Na_2_SO_4_). The herbs were purchased from a traditional Chinese medicinal store in Taipei, Taiwan, and all of the materials were authenticated by Assoc. Prof. H.C. Chang, National Laboratories of Food and Drugs, Department of Health, Executive Yuan, Taipei, Taiwan. Voucher specimens (No. RP-0001, MO-0001, CA-0001, GU-0001 and M-0001) were deposited at the Herbarium of the College of Pharmacy, Taipei Medical University. TCCT, XCCT and TWCCT were prepared according to the Unified Formula published by the Committee on Chinese Medicine and Pharmacy of the Department of Health in Taiwan (Table 3). TCCT, XCCT and TWCCT were immersed in 660, 560 and 600 mL of distilled water, respectively. Thirty grams of rhubarb was immersed in 600 mL of distilled water. The materials were boiled until half of the original water remained, and then the extract was filtered and freeze-dried until further use.

### 2.2. Determination of Total Polyphenols and Total Flavonoids

The concentration of total polyphenols in the extracts was determined by the Folin-Ciocalteu method, according to the previously described methods but with slight modifications [28]. Briefly, a diluted sample (100 mL) was added to a Folin-Ciocalteu reagent (500 mL) and 7.5% sodium carbonate solution (400 mL) and incubated at 30°C for 5 min. This mixture was then allowed to cool down in water. The absorbance was measured at 600 nm with an ELISA reader. The results are expressed as gallic acid equivalents.

The concentration of total flavonoids in the extracts was determined by using a vanillin reagent, according to previously described methods but with slight modifications [29]. Briefly, a diluted sample (300 mL) was added to a vanillin-H_2_SO_4_ solution (600 mL) at 20°C for 15 min. The absorbance was measured at 530 nm with an ELISA reader. The results are expressed as catechin equivalents.

### 2.3. Chromatographic Analysis of Herbal Preparations

The high-performance liquid chromatography (HPLC) system consisted of a Shimadzu (Kyoto, Japan) LC-10ATvp liquid chromatograph equipped with a DGU-14A degasser, an FCV-10ALvp low-pressure gradient flow control valve, an SIL-10ADvp auto injector, an SPD-M10Avp diode array detector, and an SCL-10Avp system controller. Peak areas were calculated using Shimadzu Class-VP software (version 6.12 sp5).

The mobile phase was composed of (A) water with 0.05% trifluoroacetic acid (v/v) and (B) acetonitrile with gradient elution in the following sequence: 0–30 min: (A) 10%, (B) 90%; 30–40 min: (A) 20%, (B) 80%; 40–65 min: (A) 40%, (B) 60%. Solvents were filtered through a 0.45-*μ*m FP Vericel (PVDF) membrane filter from Pall Corporation (Ann Arbor, MI, USA). A LiChrospher 100RP-18 column (250 × 4 mm I.D.) and a Purospher STAR RP-18e guard column (4 × 4 mm I.D.) (Merck, Darmstadt, Germany) were used. The flow rate was 1.0 mL min^−1^ with UV absorbance detection at 280 and 254 nm. The analysis used 20 *μ*l of a sample solution. The operation was performed at room temperature 25°C). Authentic standards used to confirm (+)-catechin, sennoside A, sennoside B, rhein and glycyrrhizic acid in the extracts were obtained from Sigma (St Louis, MO, USA). Quantification of the aforementioned compounds was expressed in micrograms per gram (*μ*g g^−1^) of extracts and was determined by a standard curve from a plot of the peak area and matching concentrations of the standard solution.

### 2.4. Experimental Animals

Male Sprague-Dawley rats (307 ± 30 g) and male ICR mice (20 ± 2 g) were obtained from the National Laboratory Animal Breeding and Research Centre (Taipei, Taiwan), and maintained in plastic cages at 21 ± 2°C with free access to water. They were kept on a 12-h light-dark cycle. Animal care and use protocol was reviewed and approved by the Institutional Animal Care and Use Committee (IACUC, Approval No: LAC-95-0026). The regulations set by IACUC met international laws and regulations.

### 2.5. Triton WR 1339-Induced Hyperlipidemia

**Figure 1:**
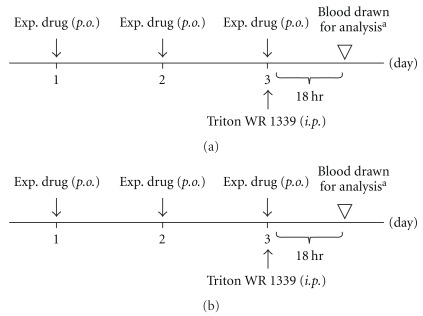
Experimental flow chart for the Triton WR 1339 experiment (a) and high-fat diet experiment (b). ^a^Blood drawn via orbital veins for TC and TG analysis. ^b^Blood drawn via tail veins for first TC, TG analysis. ^c^Blood drawn via tail veins for second TC, TG and Cr, GPT analysis.

Male ICR mice were fed a commercial diet and water ad libitum, and were kept in cages for observation for 7 days prior to the experiment. Animals were divided into seven groups of five mice each: a blank group, a control group and five experimental groups. The experimental groups received additional daily oral feeding of aqueous extracts of TCCT, XCCT, TWCCT or *R. palmatum* (200 mg kg^−1^), or fenofibrate (65 mg kg^−1^, as positive control) once a day for 3 days. An intraperitoneal injection of Triton WR-1339 (100 or 400 mg kg^−1^; Tyloxapol, Sigma, USA) was administered to all the animals except those in the blank group, an hour after the last dose of extract or drug was given. Eighteen hours after the injection and under light anesthesia, blood was drawn from the orbital vein of all the animals to measure serum total cholesterol (TC) and triglyceride (TG) with a FUGI DRI-CHEM 3500i analyser (Fuji Film, Japan), according to the manufacturer's instruction. The experimental procedure for the Triton WR 1339 experiment is shown in Figure 1(a).

### 2.6. Diet-Induced Hyperlipidemia and Obesity

Hyperlipidemia and obesity developed in animals fed on a high-fat diet. In this experiment, the male Sprague-Dawley rats were divided into six groups of six rats each; a blank group was fed a regular diet, a control group was fed a high-fat diet, and four experimental groups were fed the high-fat diet supplemented with daily administration of aqueous extracts of TCCT, XCCT, TWCCT or *R. palmatum* (200 mg kg^−1^) once by intragastric gavage. The ingredients of the high-fat and regular diets contained equal amounts of casein (14%), dextrin (15%), sucrose (10%), soybean oil (4%), cellulose (5%), mixed minerals (4%) and mixed vitamins (1%). The high-fat diet contained additional 4% lard, 0.3% cholesterol, 0.03% choline and 0.03% cholic acid. Corn starch comprised 46% of the standard rat chow diet and 42% of the high-fat diet. All the diets were prepared weekly and stored at 4°C. During the experimental period (28 days), body weight and food intake were recorded daily. Under light anesthesia, blood was drawn via the tail vein on days 14 and 28 to monitor changes in serum lipids as well as the kidney and liver functions. Biochemical analysis was performed with a FUGI DRI-CHEM 3500i analyser (Fuji Film, Japan) according to the manufacturer's instruction. The experimental procedure for the high-fat diet experiment is shown in Figure 1(b).

### 2.7. Statistical Analysis

Data were first statistically assessed by the Kruskal-Wallis one-way analysis of variance by rank. Differences between the drug-treated groups and the control group were then evaluated by the Mann-Whitney test. The differences were considered to be statistically significant at the *P* < .05. All data were expressed as the mean ± standard deviation (SD).

## 3. Results

### 3.1. Total Polyphenols and Flavonoids in the Extracts

Concentrations of total polyphenols in the extracts of TCCT, XCCT, TWCCT, and *R. palmatum* used in the experiment were in the order of *R. palmatum* > XCCT > TWCCT > TCCT. Concentrations of total flavonoids in these extracts also exhibited similar order as described in Table 3.

### 3.2. Concentrations of Major Compounds in the Extracts

The major compounds in TCCT, XCCT, and TWCCT were analyzed and quantified with HPLC. Concentrations of (+)-catechin, sennoside A and sennoside B in each decoction were in the order of *R. palmatum* > XCCT > TCCT > TWCCT. The concentration of rhein was in the order of *R. palmatum* > XCCT > TWCCT > TCCT (Table 3). Since TWCCT was the only extract containing *G. uralensis*, glycyrrhizic acid was present only in TWCCT.

### 3.3. TWCCT Lowered the Serum Triglyceride Level in Mice Treated with Triton WR 1339

**Figure 2:**
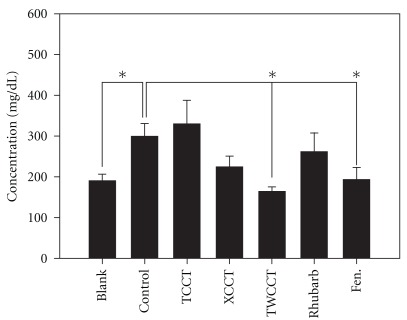
Effects of TCCT, XCCT and TWCCT on serum triglycerides in Triton WR 1339 (100 mg kg^−1^)-treated mice. Bars represent the mean ± SD. *P < .05.

Eighteen hours after the intraperitoneal injection of 100 mg kg^−1^ Triton WR-1339, the change in serum TG level differed significantly (*P* < .05). As shown in Figure 2, pair-wise comparisons between the different extract-treated and vehicle-treated animals revealed a statistically significant elevated serum TG level in the control group as compared to the blank group (*P* < .05). TWCCT pretreatment significantly reduced the serum TG level as compared to the control group (*P* < .05). Finofibrate pretreatment also showed a significant reduction of serum TG (*P* < .05).

Eighteen hours after the intraperitoneal injection of 400 mg kg^−1^ Triton WR-1339, the serum TC level in the control group showed a significant elevation as compared to the blank group (*P* < .05). However, none of the pretreatments reduced the TC level significantly.

### 3.4. XCCT and TWCCT Lowered the Lipid Profile in Rats on a High-Fat Diet

**Figure 3:**
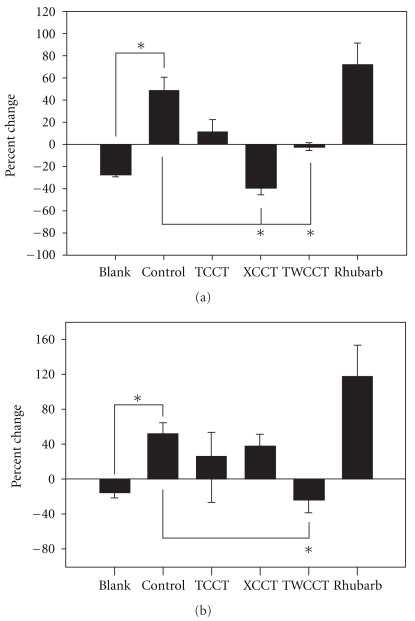
Effects of daily feeding of TCCT, XCCT and TWCCT on serum cholesterol (a) and triglycerides (b) in rats fed the high-fat diet. Bars represent the mean ± SD. *P < .05. Percent of change calculated as = ((data of week 4 – data of week 2)/data of week 2) × 100.

The effect of high-fat feeding on lipid profile over a 28-day period, with or without co-feeding the experimental herbal extracts, was examined by the percentage of change in serum TC and TG levels, obtained from blood drawn on the 14th and 28th days of the experiment. The changes in both serum TC and TG levels were significant (*P* < .05). As shown in Figure 3(a), pair-wise comparisons revealed statistically significant elevated serum TC level in the control group as compared to the blank group (*P* < .05).

XCCT or TWCCT co-treatment significantly reduced the serum TC level as compared with the control group (*P* < .05 in both the cases). The high-fat diet also induced significant elevation of TG level (*P* < .05; Figure 3(b)). Daily TWCCT co-treatment significantly attenuated the elevated TG concentration associated with high-fat diet consumption (*P* < .05; Figure 3(b)).

### 3.5. TCCT, XCCT and TWCCT Did Not Affect Body Weight, Food Intake and Feed Efficiency in Rats on a High-Fat Diet

The effects of high-fat feeding with or without co-feeding the experimental herbal extracts on body weight change, food intake and feed efficiency (FE%) were also examined. As shown in Table 4, the variations in body weight and food intake were significant (*P* < .05 in both the cases). Pair-wise comparisons revealed statistically significant elevated body weight and food intake in the control group as compared with the blank group (*P* < .05 in both the cases), and statistically significant reduced food intake in the *R. palmatum* co-treated group as compared to the control group (*P* < .05).

### 3.6. TCCT, XCCT, TWCCT and Did Not Affect Renal and Hepatic Functions in Rats on a High-Fat Diet

In order to evaluate possible renal and hepatic toxicity resulting from chronically feeding the animals on these herbal preparations, we examined the serum creatinine and GPT concentrations, obtained from blood drawn on the 28th day of the experiment. In all the experimental groups, there was no significant change in either serum creatinine or GPT level (data not shown). In addition, none of the animals experienced diarrhea during the experimental period.

## 4. Discussion

**Figure 4:**
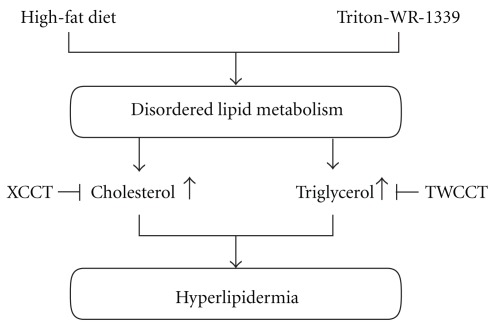
Proposed action of hypolipidermic purgatives on lipid metabolism.

**Figure 5:**
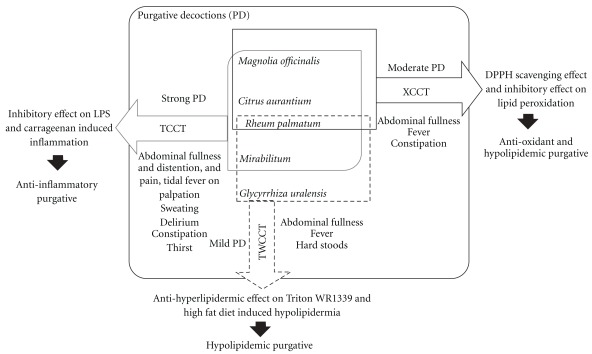
Integration of three ancient purgative formulas with modern language and science.

Modernization of TCM theory by using modern science technology and modern medicine theory is essential for the integration of old wisdom with modern medical language. This strategy and policy to integrate ancient medical knowledge with modern technology and medicine has been advocated in both Oriental and Western societies [30, 31]. According to *Shan Han Lun*, when the evil heat binds internally, many symptoms and signs may be produced, such as abdominal fullness and pain, constipation, thirst, even fever and delirium in severe cases [2, 32]. The traditional principle of treatment is to purge the evil internal heat with purgatives such as the three prescriptions used in the present study, based on the severity of symptoms [33]. In the present study, we measured the anti-obesity and hypolipidemic activities of these three decoctions with two different prevailing and relevant and *in vivo* models. TWCCT treatment not only decreased the Triton-induced hypertriglycemia, but also significantly reduced the elevated TG concentration induced by high-fat diet consumption. XCCT was the other potential lipid-modifying purgative preparation in this study. A high serum cholesterol level induced by high-fat diet was inhibited significantly by XCCT co-treatment. The results suggest the potential beneficial effects of TWCCT and XCCT in managing hypertriglycemia. A hypothetical diagram explaining the mechanism of TWCCT and XCCT-related hypolipidemic activity is shown in Figure 4. Previously, we used LPS-stimulated RAW264.7 murine macrophages and a carrageenan-induced paw edema in mice to show that TCCT has a potent anti-inflammatory effect in addition to its traditionally known strong purgative activity. The result suggests that TCCT may be indicated to alleviate inflammatory conditions in patients with concomitant constipation [26]. Next, we used well established models to assay the antioxidant activity in 3 CCTDs *in vivo* or *in vitro*, namely, 1,1-diphenyl-2-picrylhydrazyl (DPPH) radical scavenging test; measurement of malonedialdehyde (MDA) level in Butylhydroperoxide (TBH); and CCl_4_-induced lipid peroxidation. The results of that study suggest that XCCT has a significant antioxidant activity and hepatic protection potential [27]. We developed a flowchart diagram to explicate our work on the translation of these ancient formulas into modern language, shown in Figure 5.

To the best of our knowledge, this is the first report of comparing the lipid-modifying effects of these three purgative formulations with fenofibrate, a third-generation fibric acid derivative, synthesized in 1975 [34]. Triton-induced hypertriglycemia was similarly lowered by pretreatment with the TWCCT extracts (about 45%) and fenofibrate (about 35%), shown in Figure 2. The result suggests that the mechanism of action of TWCCT on lipid metabolism may be similar to that of fenofibrate. Fibrates usually reduce plasma levels of triglycerides by 30–50%, attributable to the activation of the nuclear transcription factor peroxisome proliferator-activated receptor alpha (PPAR*α*). The activation of PPAR*α* then alters the transcription rate of target genes associated with lipid metabolism and inflammatory response, such as lipoprotein lipase, *β*-oxidation pathway, and COX-2 [35]. *R. palmatum* is the only common constituent in XCCT and TWCCT, and also the principle herb in these two preparations by TCM. Lipids are metabolized in several tissues, including adipose tissue, liver, intestine and muscles. Rhubarb extracts have been found to enhance gastrointestinal transit and contractility [9, 36]. Rhubarb extracts also enhance bile excretion and inhibit the activity of pancreatic lipase, HMG-CoA, and squalene epoxidase [11, 37]. Sennoside B and rhein reduce the differentiation of adipocytes and the accumulation of triglyceride within cells [10]. Dietary products rich in flavonoids and polyphenols have been reported to correlate with lower plasma TC concentrations and plasma LDL cholesterol concentrations in humans [38–41]. In the chromatographic analysis, we identified the existence of significant amounts of polyphenols, flavonoids, and compounds such as catechin, sennosides and rhein in the extracts of our experiments. Major compounds found in citrus, magnolia, and licorices also have shown serum lipid-lowering effects [42–44]. Together, these may contribute to the hypolipidemic effect of TWCCT and XCCT. However at present, the chemical constituents identified in this experiment could be used as reference standards to ensure the quality of herbal products and the validity of the comparison between studies using the same product. However, the precise mechanism by which TWCCT and XCCT influence the serum TG and TC responses remains to be determined.

In our study, the co-treatment of *R. palmatum* extract significantly reduced the food intake of the high-fat diet animals, but it did not reduce their body weight significantly. We also demonstrated that at the particular dosage used in the experiment, rhubarb did not reduce the serum lipid level of animals fed with a high-fat diet. In high-fat feeding experiment, higher FE was an indicator of the ease with which animals gain weight from what they eat [45]. All experimental and control groups showed higher feed efficiencies than the blank group. This result suggests that these purgatives have no anti-obesity effect. Previous studies also demonstrated that laxatives are either ineffective weight-reducing agents, or they induce adverse events [5, 46–48]. Therefore, clinicians should be discouraged in using these purgative preparations for the purpose of weight reduction.

## 5. Conclusions and Directions for Future Research

In conclusion, the results suggest that TWCCT and XCCT might exert beneficial effects in the management of hyperlipidemia. Further mechanistic and clinical studies are needed to confirm the clinical usefulness of TWCCT and XCCT.

## Funding

Committee on Chinese Medicine and Pharmacy, Department of Health, Executive Yuan, Taiwan, (grant no. CCMP92-CT-09) and Taipei Medical University (grant no. TMU97-AE1-B11).

## Figures and Tables

**Table 1:** Summary of studies examining the metabolic effect of rhubarb or rhubarb-containing polyherbal preparations on body weight, serum TC level, and TG level in animal models.

Ref.	Animal	Stress	Duration	Treatment	Metabolic effect		
					Body weight^a^	TC^b^	TG^c^
[7]	C57BL/6J mice	Cholesterol diet	4 weeks	*Rheum rhaponticum* stalk fibre (50 g kg^−1^)	NSD (greater weight gain in T group)	↓*	NR
[8]	Diabetic BBdp rats; SD rats	STZ (for SD rats)	2 weeks	*Rheum rhaponticum* stalk fibre (50 g kg^−1^)	NE (greater weight gain in STZ group)	NSD	NSD
[9]	Wistar rats	Alloxan/ gastroparesis	Once	Water extracts of *Rheum palmatum* (150–300 mg kg^−1^), oral	NR	NR	↓* (Postprandial)
[10]	C57BL6J mice	STZ	8 weeks	70% EtOH rhubarb extracts (5 mg kg^−1^)	↓*	NR	NR
[11]	SD rats	Triton WR-1339	3 days	DOT (200 mg kg^−1^)	NR	↓*	↓*
[12]	Wistar rats	High-fat diet	8 weeks	NT	↓*	NSD	NSD
[13]	ICR mice	High-fat diet	14 weeks	IN, BO or DA (31.5 mg) mixed in diet	↓* in DA and BO group (body growth)	↓* in DA and BO group	NE (inhibition noted but not significant)
[14]	Beagle dogs	None	4 and 13 weeks	ERr731	NSD	NR	NR

NSD, no significant difference; ↓, reduced; NR, not reported in the study. T, treatment (experimental) group; STZ, streptozotocin; DOT, Daio-Orengedokuto, consists of Rhei Rhizoma, Scutellariae Radix, Coptidis Rhizoma, Phellodendri Cortex and Gardeniae Fructus; IN, Yin-chen-hao-tang, consists of Rhei Rhizoma, Artemisiae Capillaris Flos, Gardeniae Fructus; BO, Bofu-tsusho-san, consists of Rhei Rhizoma, Scutellariae Radix, Glycyrrhizae Radix, Platycodi Radix, Atractylodis Lanceae Rhizoma, Schizonepetae Spica, Gardeniae Fructus, Paeonia Radix, Cnidii Rhizoma, Angelicae Radix, Menthae Folium, Saposhnikoviae Radix, Ephedrae herba, Forsythiae Fructus, Zingiberis Rhizoma, Kaolinum, Gypsum Fibrosum, Natrium Sulfuricum; DA, Dai-saiko-to, consists of Rhei Rhizoma, Bupleuri Radix, Pinelliae Tuber, Scutellariae Radix, Paeonia Radix, Zizypus Fructus, Aurantii Fructus Immaturus, Zingiberis Rhizoma. (DOT, IN, BO, and DA are all rhubarb-containing polyherbal preparations). NT, contains 40% rhubarb root and stem; ERr 731, special dry extract from the roots of *R. rhaponticum*.

^a^ Effect on body weight reduction; ^b^ on TC reduction; ^c^ Effect on TG reduction; * Statistically significant.

**Table 2:** Summary of human clinical trials examining the metabolic effects of rhubarb or rhubarb-containing polyherbal preparations on body weight, serum total cholesterol (TC) and triglyceride (TG).

Ref.	Study type	Subjects	Treatment^c^	Duration	Metabolic effect			
					Body weight^a^	TC^b^	TG^c^	Adverse effects
[15]	Case series	10 men with hyper-cholesterolemia	Fibre of *Rheum rhaponticum* (27 g)	4 weeks	NSD	↓* (8%)	↓	none
[16]	R, case- control	95 pregnancy induced hypertensive women	Diet control and Western medication with or without prepared rhubarb (3–9 g)	6–8 weeks	NR	T: NSD	T: ↓*	NR
[17]	Case series	232 NAFLD patients	Danning Pian	3 months	NSD	NSD	↓*	15.1% including diarrhoea [32], itchy skin [1], nausea [3], increased ALT [2]
[18]	R, db, pc	81 obese female with impaired glucose tolerance	Diet and exercise with either Bofu-Tsusho-San (BO) or placebo	24 weeks	P: ↓* (7.6%); T: ↓* (11.8%)	P: ↓*; T: ↓*	P: ↓*; T: ↓*	3 in BO group dropped out due to noncompliance because of loose stool
[19]	R, db, pc	110 perimenopause women	ERr731 or placebo	12 weeks	P:NSD T:NSD	NR	NR	No
[20]	R, case-control	24 healthy women	Diet control with NT (250–500 mg) or placebo	12 weeks	P: ↓, T:NSD	NR	NR	Dose-dependent soft stool in T group
[21]	R, P-control	105 healthy volunteers	Low or high dose of mixture of NT with gallic acid or placebo	24 weeks	P: ↓, T(low dose): ↓; T (high dose): ↑	NSD	NSD	40% cases dropped out due to ineffectiveness
[22]	R, db, control	135 NAFLD patients	T: Danning Pian	24 weeks	C:NE; T: ↓*	C: ↓; T: ↓	C: ↓*; T: ↓*	T: most showed diarrhoea, skin rash [1], nausea [3]

NSD, no significant difference; ↓, reduced; NR, not reported in the study; ↑, increased. R, randomized; db, double-blind; pc, placebo-controlled; P, placebo group; T, treatment (experimental) group; C, control group. NAFLD, non-alcoholic fatty liver disease; ERr 731, extract from the roots of Rheum rhaponticum; NT, contains 40% rhubarb root and stem; Danning Pian: each tablet contain 4.5 g Radix et Rhizoma Rhei. BO, Bofu-tsusho-san, consists of Rhei Rhizoma, Scutellariae Radix, Glycyrrhizae Radix, Platycodi Radix, Atractylodis Lanceae Rhizoma, Schizonepetae Spica, Gardeniae Fructus, Paeonia Radix, Cnidii Rhizoma, Angelicae Radix, Menthae Folium, Saposhnikoviae Radix, Ephedrae herba, Forsythiae Fructus, Zingiberis Rhizoma, Kaolinum, Gypsum Fibrosum, Natrium Sulfuricum.

^a^ Effect on body weight reduction; ^b^ Effect on TC reduction; ^c^ Effect on TG reduction; * Statistically significant.

**Table 3:** Constituents and chemical profiles of TCCT, XCCT, and TWCCT.

Formula name	Constituent	Weight (g)	Yield (%)	Catechin (*μ*g g^−1^)^a^	Sennoside B (*μ*g g^−1^)^a^	Sennoside A (*μ*g g^−1^)^a^	Rhein (*μ*g g^−1^)^a^	Glycyrrhizic acid (*μ*g g^−1^)^a^	Total polyphenols (*μ*g mg^−1^)^b^	Total flavonoids (*μ*g mg^−1^)^c^
	*R. palmatum*	8	27.3	3025.8 ± 90.5	3214.2 ± 106.1	1815.4 ± 60.8	251.2 ± 19.0	—	110.3 ± 1.1	24.4 ± 0.7
TCCT	*M. officinalis*	16								
	*C. aurantium*	3								
	*Mirabilitum*	6								
	*R. palmatum*	14	19.3	5828.1 ± 21.0	3945.6 ± 10.9	2458.0 ± 18.0	657.6 ± 17.5	—	11.5 ± 10.9	123.8 ± 0.4
XCCT	*M. officinalis*	7								
	*C. aurantium*	7								
	*R. palmatum*	12	31.9	2904.6 ± 45.1	1998.0 ± 125.1	1565.8 ± 119.6	382.7 ± 9.70	336.8 ± 51.0	117.5 ± 12.5	40.1 ± 1.7
TWCCT	*Mirabilitum*	12								
	*G. uralensis*	6								
	*R. palmatum*	30	12.21	6729.1 ± 163.1	4024.6 ± 121.5	3170.0 ± 96.2	681.3 ± 20.7	—	255.5 ± 14.0	165.9 ± 3.7

^a^ The amount is expressed as *μ*g g^−1^ of extract; ^b^ The content is expressed as *μ*g of gallic acid equivalent per mg of extract; ^c^ The content is expressed as *μ*g of catechin equivalent per mg of extract.

**Table 4:** Changes in body weight, food intake, and feed efficiency in rats fed different diets.

	Blank	Control	TCCT	XCCT	TWCCT	Rhubarb
Daily weight change (g)	2.5 ± 1.0	4.2 ± 0.6*	3.5 ± 0.7	4.3 ± 0.5	4.3 ± 0.4	3.9 ± 1.1
Daily food intake (g)	21.0 ± 1.4	22.0 ± 1.3*	20.8 ± 1.3	23.0 ± 1.3	22.7 ± 0.5	20.3 ± 1.8*
Feed efficiency (%)	12.3 ± 4.5	18.6 ± 2.9	17.0 ± 3.6	18.6 ± 2.9	19.1 ± 2.0	19.2 ± 5.2

^∗a^ *P* < .05.
